# Wild edible fool’s watercress, a potential crop with high nutraceutical properties

**DOI:** 10.7717/peerj.6296

**Published:** 2019-02-01

**Authors:** Carla Guijarro-Real, Jaime Prohens, Adrian Rodriguez-Burruezo, Ana María Adalid-Martínez, M Pilar López-Gresa, Ana Fita

**Affiliations:** 1 Instituto de Conservación y Mejora de la Agrodiversidad Valenciana (COMAV), Universitat Politècnica de València, Valencia, Spain; 2 Instituto de Biología Molecular y Celular de Plantas (IBMCP), Universitat Politècnica de València, Valencia, Spain

**Keywords:** Antioxidants, *Apium nodiflorum*, DPPH, New crops, Total phenolics, Quercetin, Wild edible plants, Flavonoids

## Abstract

**Background:**

Fool’s watercress (*Apium nodiflorum*) is an edible vegetable with potential as a new crop. However, little information is available regarding the antioxidant properties of the plant and the individual phenolics accounting for this capacity are unknown.

**Methods:**

The antioxidant properties of twenty-five wild populations were analysed and individual phenolics present in the species reported and compared with celery and parsley. The antioxidant activity was measured as the 2,2-diphenyl-1-picrylhydrazyl hydrate (DPPH) free radical scavenging capacity, and the total phenolics content (TPC) via the Folin-Ciocalteu procedure. The individual phenolics constituents were determined via high performance liquid chromatography (HPLC) as aglycones.

**Results:**

The average DPPH and TPC of fool’s watercress were 28.1 mg Trolox g^−1^ DW and 22.3 mg of chlorogenic acid equivalents g^−1^ DW, respectively, much higher than those of celery and parsley. Significant differences for both DPPH and TPC, which may be explained by either genotype or environmental factors, were detected among groups established according to geographical origin. Quercetin was identified as the major phenolic present in the leaves of the species, unlike parsley and celery, in which high amounts of apigenin and luteolin were determined. Quercetin represented 61.6% of the phenolics targeted in fool’s watercress, followed by caffeic acid derivatives as main hydroxycinnamic acids.

**Discussion:**

The study reports the high antioxidant properties of fool’s watercress based on a large number of populations. Results suggest that quercetin accounts for an important share of the antioxidant capacity of this potential new crop. The study also provides a basis for future breeding programs, suggesting that selection by geographical locations may result in differences in the antioxidant properties.

## Introduction

Wild fruits and vegetables are part of the traditional cuisine in many countries of the Mediterranean region. Besides enriching the cuisine with particular tastes, many of them have also been used in the past as dietary supplements or sources of bioactive compounds, as well as in traditional medicine (Shikov et al., 2017). In the last decades, there has been an increasing interest in wild vegetables by consumers. Consequently, several works have evaluated the nutritional value of wild edible species and also assessed their bioactive health promoting properties (Motamed & Naghibi, 2010; Egea-Gilabert et al., 2013; García-Herrera et al., 2014). Moreover, domestication of wild species to be grown as new crops is an opportunity for increasing the offer in food markets. As examples, salad rocket (*Eruca sativa* Mill. and *Diplotaxis tenuifolia* (L.) DC.) and watercress (*Rorippa nasturtium-acquaticum* Hayek) have been adapted and developed as common crops (Molina, Pardo-de Santayana & Tardío, 2016).

*Apium nodiflorum* (L.) Lag., commonly known as fool’s watercress or water celery, is a perennial herb from *Apiaceae* family. Well adapted to damp soils, it can be easily found, forming clamps, in fresh, shallow water courses such as streams or ditches. The species is broadly distributed along the temperate areas of central and southern Europe, northern Africa and western and central Asia (Tardío et al., 2016). It is widely distributed in Spain, including the Mediterranean coast (Knees, 2003), a region with an ancient agricultural tradition. However, the alteration in the irrigation system to drip irrigation in agriculture and the reduction of river flows may negatively affect its natural distribution.

Wild fool’s watercress has been traditionally harvested and consumed in several Mediterranean countries, such as Spain, Italy, Portugal or Morocco (Tardío et al., 2016). The edible parts are the young leaves and tender shoots, which are used as a vegetable and mainly consumed raw in salads, or to a lesser extent boiled or included as a condiment in soups and other dishes (Parada, Carrió & Vallès, 2011; Guarrera & Savo, 2016). The species has been reported as appetite enhancer, diuretic, intestinal anti-inflammatory, antimicrobial and antifungal (Menghini et al., 2010; Maxia et al., 2012; Guarrera & Savo, 2013; Tardío et al., 2016). However, the nutritional and bioactive value of the species has not been extensively studied. García-Herrera (2014) classified fool’s watercress as a vegetable with a high content in calcium and sodium, although its consumption should be moderate for people with kidney damage due to the content in oxalic acid, as revealed by Morales (2011). The plant may be also considered as a source of vitamin E and B_9_ (Tardío et al., 2016). But the greatest interest considering its nutritional capacity is probably due to the high content in phenolic compounds together with the strong antioxidant activity that presents (Morales et al., 2012).

Phenolic compounds can be included into different categories attending to their chemical composition, being flavonoids and phenolic acids the most common classes in plants (Zhou et al., 2016). They commonly appear as glycosides in plants, conjugated to other molecules such as sugars, amines, organic acids or other phenolic compounds (Barba, Esteve & Frígola, 2014). Besides the importance of these metabolites for plants defence and survival (Cartea et al., 2011), flavonoids and phenolic acids are considered of great importance for human health due to their antioxidant capacity (Kaushik et al., 2015; Sahidi & Ambigaipalan, 2015). As antioxidants, they neutralize reactive oxygen species, which in excess can produce molecular and cellular disorders causing several diseases (Prasad, Gupta & Tyagi, 2017). However, this capacity is greatly dependent on the chemical structure of each molecule (Zaluski, Ciesla & Janeczko, 2015).

Flavonoids and phenolic acids are commonly found in *Apiaceae* (Sayed-Ahmad et al., 2017) and daily used spices and aromatic herbs of the family have been studied for these compounds. For instance, leaves of parsley (*Petroselinum crispum* (Mill.) Nyman) and celery (*Apium graveolens* L. var. *dulce*) are good sources of apigenin (Pápay et al., 2016; Zhou et al., 2017), with levels that can reach 630 mg 100 g^−1^ FW (Justesen & Knuthsen, 2001) and 970 mg 100 g^−1^ FW (Yao & Ren, 2011), respectively. Polyphenol glycosides including apigenin, quercetin, chlorogenic acid, caffeic acid or ferulic acid derivatives have been detected in fennel (*Foeniculum vulgare* Mill.) (Salami, Rahimmalek & Ehtemam, 2016). And Barros et al. (2012) determined that coriander leaves (*Coriandrum sativum* L.) are rich in quercetin derivatives, with a total value of 494 mg 100 g^−1^ DW, and also present relevant contents of *p*-coumaric acid derivatives. However, we have not found references to the phenolic constituents of the edible organs of fool’s watercress.

We consider that there is a need to evaluate the antioxidant properties and phenolic composition of fool’s watercress since this edible species has potential as a source of antioxidants. So far, little information on the diversity for phenolics content and antioxidant activity in the species is available (Morales et al., 2012). The study of several populations may offer more accurate information for the antioxidant properties and phenolic content of the species. Thus, in the present study we evaluated the antioxidant activity of a set of populations of fool’s watercress. We also determined the main phenolic compounds in an attempt to correlate them with the antioxidant properties of this species. We included two related crops with similar uses in the analysis (celery and parsley) in order to compare data of wild and related cultivated species. The results obtained may be also useful for considering the domestication of fool’s watercress.

## Materials and Methods

### Plant material and sample preparation

**Figure 1:**
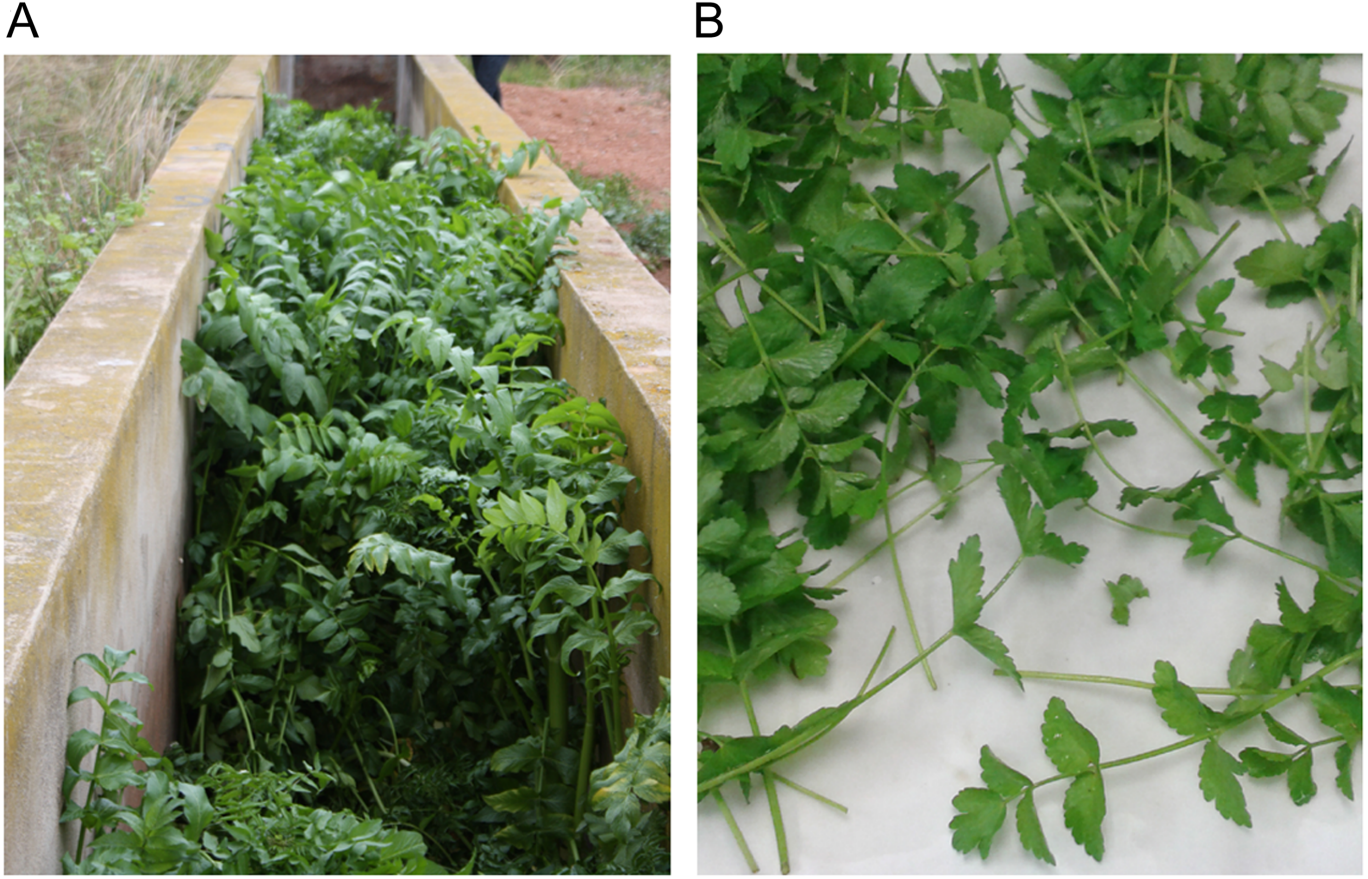
Plant of fool’s watercress. (A) Wild population growing in an irrigation ditch. (B) Sample representing the edible part of this plant. Author: C. Guijarro-Real

The Horta Nord of Valencia (Spain), an area with many irrigation ditches used for centuries by the farmers, was prospected. The prospection took place in the spring season of 2015 and was focused on the locations where ditches are still in use and a regular water flow is provided (Fig. 1). A total of twenty-five wild isolated masses of fool’s watercress were sampled. Samples were grouped by their geographical origin and seven groups were established according to the following geographical areas: Puerto de Sagunto (FW1), Puzol-El Puig (FW2), Masamagrell (FW3), Albuixech-Albalat dels Sorells (FW4), Foios-Meliana town (FW5), Meliana beach-Alboraya-Valencia (FW6) and Pueblo Nuevo-Alfara del Patriarca (FW7) (Table 1).

**Table 1:** Geographical situation of the wild populations of fool’s watercress harvested in the region of Valencia (Spain) and identification of the groups established by their origin.

**Geographical group**^a^	**Population**^b^	**Location**	**Coordinates**	
FW1	Nod-001	Puerto de Sagunto	39°37′36″N	0°16′49″W
	Nod-002	Puerto de Sagunto	39°37′55″N	0°16′11″W
FW2	Nod-003	Puzol	39°36′08″N	0°18′08″W
	Nod-004	El Puig	39°35′09″N	0°17′45″W
	Nod-005	El Puig	39°36′00″N	0°18′05″W
	Nod-006	El Puig	39°35′33″N	0°19′16″W
FW3	Nod-007	Masamagrell	39°34′04″N	0°19′42″W
	Nod-008	Masamagrell	39°33′59″N	0°19′48″W
	Nod-009	Masamagrell	39°33′42″N	0°18′25″W
FW4	Nod-010	Albuixech	39°33′04″N	0°19′39″W
	Nod-011	Albuixech	39°32′37″N	0°19′55″W
	Nod-012	Albuixech	39°32′29″N	0°19′27″W
	Nod-013	Albuixech	39°32′43″N	0°19′07″W
	Nod-014	Albalat dels Sorells	39°32′04″N	0°19′26″W
FW5	Nod-015	Foios	39°31′59″N	0°20′31″W
	Nod-016	Foios	39°32′16″N	0°20′49″W
	Nod-017	Meliana (town)	39°31′26″N	0°20′52″W
FW6	Nod-018	Meliana (beach)	39°31′01″N	0°19′36″W
	Nod-019	Alboraya	39°30′59″N	0°19′37″W
	Nod-020	Valencia	39°28′57″N	0°20′11″W
FW7	Nod-021	Pueblo Nuevo	39°30′39″N	0°22′57″W
	Nod-022	Pueblo Nuevo	39°30′39″N	0°23′10″W
	Nod-023	Pueblo Nuevo	39°31′19″N	0°23′17″W
	Nod-024	Alfara del Patriarca	39°31′37″N	0°22′36″W
	Nod-025	Alfara del Patriarca	39°32′21″N	0°23′06″W

**Notes.**

a Codes FW1 to FW7 refer to the seven geographical groups in which the populations of fool’s watercress have been clustered.

b Nod-001 to Nod-025 refer to the codes given to the twenty-five populations of fool’s watercress analysed in the study.

The aerial part was air-dried in oven at 37 °C, with low humidity conditions, for three days, in order to prevent water activity (Fig. 1). Dried samples were powdered with a commercial grinder and used for the determinations, which were carried out in triplicates. For comparison of results, celery and parsley species were also analysed. Thus, two commercial samples of celery, and two commercial samples of parsley, each one coming from different local markets, were acquired and processed in the same way than samples of fool’s watercress.

### Evaluation of the antioxidant activity and total phenolics content

The antioxidant activity was measured as the 2,2-diphenyl-1-picrylhydrazyl hydrate (DPPH) free radical scavenging capacity as described by Rufino et al. (2007). Subsamples of 0.1 g were extracted with 5 mL methanol (50% v/v) plus 5 mL acetone (70% v/v), then samples of fool’s watercress diluted (1:2). Absorbance was measured at 515 nm after 25 min of incubation with DPPH (Sigma-Aldrich, Sant Louis, MO, USA) solution (0.025 g/L in methanol). The antioxidant Trolox (Scharlab S.L., Sentmenat, Barcelona, Spain) was used as standard and results were expressed as milligrams of Trolox equivalents per gram of dry weight (mg Trolox g^−1^ DW).

Total phenolics were extracted from subsamples of 0.125 g with 5 mL acetone (70% v/v) containing glacial acetic acid (0.5% v/v) and determined according to the Folin-Ciocalteu procedure (Singleton & Rossi, 1965) as indicated in Plazas et al. (2014). Absorbance was measured at 750 nm after 95 min of incubation with the diluted Folin-Ciocalteu reagent (10% v/v) (Scharlab S.L.). Chlorogenic acid (Sigma-Aldrich) was used as standard and results were expressed as milligrams of chlorogenic acid equivalents per gram of dry weight (mg CAE g^−1^ DW).

### Phenolics profile

Phenolic compounds were extracted from subsamples of 0.1 g with 1.5 mL methanol (80% v/v) including 0.1% (w/v) 2,6-di-*tert*-butyl-4-methylphenol (BHT) (Sigma-Aldrich) (Plazas et al., 2014). Then, an hydrolysis was performed by adding 1.2 M HCl for two hours at 95 °C for fool’s watercress and celery, and 2 M HCl for four hours for parsley (Justesen & Knuthsen, 2001), to a final methanol solution of 50% (v/v).

Samples were analysed on a HPLC 1220 Infinity LC System (Agilent Technologies, Santa Clara, CA, USA). A BRISA C18 column (150 mm × 4.6 mm i.d., 3 µm particle size; Teknokroma, Barcelona, Spain) was used and the injection volume was 10 µl. Mobile phase consisted of two solvents, (A) 0.1% formic acid in water and (B) methanol with gradient elution. Hydroxycinnamic acids profile was studied under the following conditions (Yildiz et al., 2008): starting with 7% (B) the first 8 min, raising up to 30% (B) at 13 min, 66% (B) at 48 min, 75% (B) at 50 min, 100% (B) at 54 min and maintained for 2 min, then decreasing to initial conditions of 7% (B) at 60 min and equilibrated for 5 min. Flow rate was 1 mL min^−1^ and the absorbance was fixed at 320 nm. The study of flavonoids was performed as described by Bae et al. (2012) with a flow rate of 0.8 mL min^−1^ and a fixed absorbance of 360 nm, using the same solvents as above. The gradient elution started with 40% (B) following to 100% (B) at 10 min and maintained for 5 min, then decreasing to the initial conditions at 20 min and equilibrated for 5 min.

**Figure 2:**
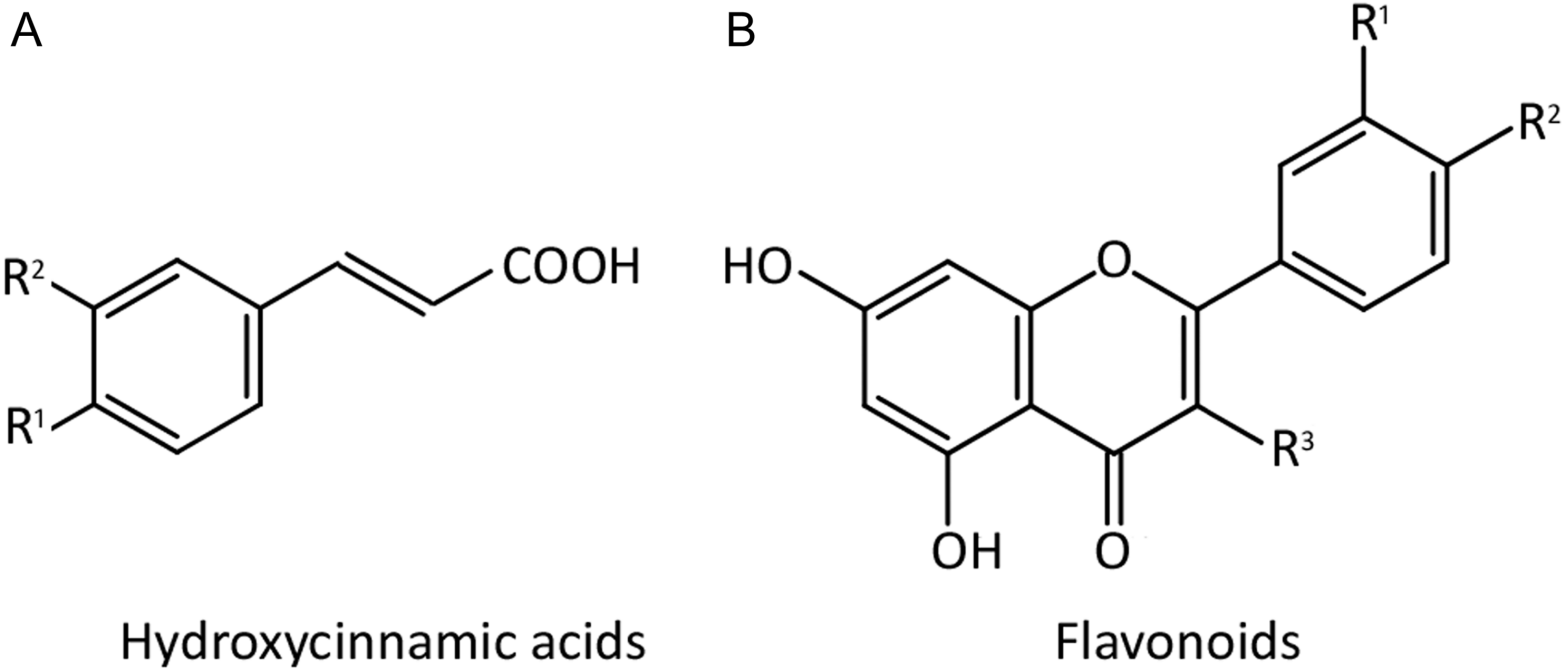
Chemical structure of the phenolic compounds evaluated in the samples of fool’s watercress, celery and parsley. The phenolics targeted included the following hydroxycinnamic acids (A): caffeic acid (*R*^1^ = *R*^2^ = OH), chlorogenic acid (*R*^1^ = *R*^2^ = OH plus the carboxylic group esterified with quinic acid), *p*-coumaric acid (*R*^1^ = OH, *R*^2^ = H) and ferulic acid (*R*^1^ = OH, *R*^2^ = OCH_3_); and the flavonoids (B): apigenin (*R*^1^ = OH, *R*^2^ = *R*^3^ = H), kaempferol (*R*^1^ = *R*^3^ = OH, *R*^2^ = H), luteolin (*R*^1^ = *R*^2^ = OH, *R*^3^ = H) and quercetin (*R*^1^ = *R*^2^ = *R*^3^ = OH).

A tentative identification of the compounds was performed by comparison of the retention time from the peaks with commercial standards (Sigma-Aldrich), and with published data. Standards from common phenolics described in *Apiaceae* were selected and used in this study (Table 2), which their general chemical structures are represented in Fig. 2. Due to the possible partial hydrolysis of chlorogenic acid to caffeic acid under the above cited conditions, concentration of both compounds were added and considered together as caffeic acid derivatives.

**Table 2:** Phenolic aglycones commonly cited in the literature for the family *Apiaceae*. It is indicated the class classification according to the chemical structure as well as the retention time in the current conditions (Rt, min).

**Rt**^a^	**Compound**	**Class**	**References**
8.0	Quercetin	Flavonol	Justesen (2000), Justesen & Knuthsen (2001), Barros et al. (2012), Vallverdú-Queralt et al., (2014) and Salami, Rahimmalek & Ehtemam (2016)
8.4	Luteolin	Flavone	Crozier et al. (1997), Justesen (2000), Justesen & Knuthsen (2001), Viña & Chaves (2007), Yildiz et al., (2008) and Barros et al. (2012)
9.0	Kaempferol	Flavonol	Justesen, Knuthsen & Leth (1998), Justesen (2000), Justesen & Knuthsen (2001), Yao et al. (2010), Yao & Ren (2011) and Barros et al., (2012)
9.2	Apigenin	Flavone	Crozier et al. (1997), Justesen & Knuthsen (2001), Yildiz et al. (2008)Hossain et al. (2011) and Barros et al. (2012)
16.3	Chlorogenic acid	Hydroxycinnamic acid	Viña & Chaves (2007), Barros et al., (2012), Vallverdú-Queralt et al. (2014) and Salami, Rahimmalek & Ehtemam (2016)
17.3	Caffeic acid	Hydroxycinnamic acid	Yao et al. (2010), Hossain et al. (2011), Yao & Ren (2011), Vallverdú-Queralt et al. (2014) and Salami, Rahimmalek & Ehtemam (2016)
20.7	*p-* Coumaric acid	Hydroxycinnamic acid	Yao et al. (2010), Yao & Ren (2011), Barros et al. (2012), Vallverdú-Queralt et al. (2014) and Salami, Rahimmalek & Ehtemam (2016)
21.9	Ferulic acid	Hydroxycinnamic acid	Yao et al. (2010), Yao & Ren (2011), Barros et al. (2012), Vallverdú-Queralt et al., (2014) and Salami, Rahimmalek & Ehtemam (2016)

**Notes.**

a Rt obtained in the conditions described by Yildiz et al. (2008) (hydroxycinnamic acids) or Bae et al. (2012) (flavones and flavonols).

### Data analysis

The average values of DPPH and total phenolics content (TPC) in each sample were used to obtain the mean value and the average standard error of the seven geographical groups of fool’s watercress (FW1-FW7), celery (GRAV, as average of two samples, Grav-01 and Grav-02), and parsley (CRI, as average of two samples, Cri-01 and Cri-02). Data were analysed using a one-way factorial analysis of variance (ANOVA) considering the groups as a factor and significant differences between groups were calculated with the Student-Newman-Keuls test. Ten selected populations of fool’s watercress with low and high antioxidant activities and overall representing four geographical groups, plus the two samples of celery and the two of parsley, were analysed for phenolic profile by HPLC, in triplicates. Finally, Pearson pairwise comparisons were performed in order to evaluate correlations between DPPH, TPC and the content in phenolics determined as sum of the individual phenolics targeted.

## Results

### DPPH radical-scavenging activity and TPC

A highly significant variation (*P* < 0.001) between fool’s watercress and celery and parsley was found for the DPPH scavenging capacity (Table 3). The average DPPH capacity of fool’s watercress was 28.12 mg Trolox g^−1^ DW. This value was 3.5-fold higher than the DPPH value for celery (8.09 mg Trolox g^−1^ DW) and 12.8-fold higher compared to parsley (2.20 mg Trolox g^−1^ DW). Values of the seven geographical groups considered in fool’s watercress ranged from 15.17 to 36.97 mg Trolox g^−1^ DW (FW2 and FW7, respectively; *P* <  0.01), with continuous variation among them (Table 3).

**Table 3:** Mean values and range for DPPH radical-scavenging activity and TPC for fool’s watercress, celery, and parsley groups. *N* is the number of populations included in each group. Statistics includes the mean squares values (MS) for group and residuals, and the value of the *F*-test for differences among groups.

		*(mg Trolox g*^−1^*DW)*		*(mg CAE g*^−1^*DW)*	
**Group**^a^	***N***	**DPPH**^b^	**Range**	**TPC**^b^	**Range**
FW1	2	28.19 cd	(22.84–33.55)	24.16 c	(23.12–25.21)
FW2	4	15.17 bc	(10.34–18.20)	15.90 ab	(13.26–18.91)
FW3	3	28.61 cd	(19.95–36.20)	23.73 c	(21.31–26.97)
FW4	5	28.29 cd	(19.86–38.22)	22.73 c	(18.58–27.56)
FW5	3	25.67 cd	(21.14–29.59)	19.82 bc	(18.55–21.82)
FW6	3	32.23 d	(30.74–33.82)	23.89 c	(23.17–24.39)
FW7	5	36.97 d	(27.96–43.51)	26.19 c	(21.19–28.91)
					
GRAV	2	8.09 ab	(7.47–8.71)	13.32 a	(12.71–13.93)
					
CRI	2	2.20 a	(1.87–2.52)	14.98 ab	(12.58–17.38)
					
*MS group*		*373.28*		*64.28*	
*MS residual*		*36.98*		*7.02*	
Prob *F-* test		<0.0001		<0.0001	

**Notes.**

a Groups FW1 to FW7 refer to the seven geographical groups in which the populations of fool’s watercress have been clustered (see Table 1). GRAV refers to the celery group, including Grav-01 and Grav-02 samples. CRI refers to the parsley group, including Cri-01 and Cri-02 samples.

b Different letters indicate significant differences according to the Student-Newman-Keuls test (confidence level 95.0%).

In the case of TPC, differences were less remarkable in absolute values but also highly significant (*P* < 0.001) (Table 3). Celery and parsley presented similar average values of 13.32 mg of chlorogenic acid equivalents (CAE) g^−1^ DW and 14.98 mg CAE g^−1^ DW, respectively, while the average content determined for fool’s watercress was 22.35 mg CAE g^−1^ DW. However, the variation in this species ranged from 15.90 (FW2) to 26.19 mg CAE g^−1^ DW (FW7), which meant that geographical groups with the lowest content were comparable to celery and parsley. As in the DPPH radical-scavenging activity, a continuous variation was observed for the total of groups established with significant differences (*P* < 0.01).

### Phenolic profile of fool’s watercress, celery, and parsley

Ten populations of fool’s watercress from diverse geographical groups (FW2, FW4, FW5 and FW7) and overall representing samples with low and high antioxidant capacity and TPC were used for analysing the phenolic profile of the species and comparing it with those of celery and parsley (Fig. S1). The content in phenolics of fool’s watercress, obtained as sum of individual phenolics, ranged from 1.20 to 7.12 mg g^−1^ DW (for populations Nod-004 and Nod-021, respectively), with a mean value of 4.19 mg g^−1^ DW (Table 4). Samples with the highest values corresponded to the geographical group FW7 (mean value 6.71 mg g^−1^ DW) while those with low content belonged to the geographical groups FW2 and FW5 (mean values 1.92 and 2.76 mg g^−1^ DW, respectively). Populations from geographical group FW4 showed intermediate content (mean value 4.15 mg g^−1^ DW). On the other hand, the mean values for celery and parsley were 7.63 and 4.76 mg g^−1^ DW, respectively.

**Table 4:** Mean values for the content of individual phenolics targeted, DPPH radical-scavenging activity and TPC in individual samples of fool’s watercress, celery, and parsley. The content of each phenolic targeted is expressed as µg g^−1^ DW, the sum of the individual phenolics targeted (Σ i.p.) as mg g^−1^ DW, the DPPH as mg Trolox g^−1^ DW and the TPC as mg CAE g^−1^ DW. Statistics includes the mean squares values (MS) for species and residuals, and the value of the *F*-test for differences among species.

	**CF der**	**CMA**	**FRA**	**AP**	**KM**	**LT**	**QR**	Σ**i.p.**	**DPPH**	**TPC**
Nod-003^a^	640.9	139.9	55.8	12.2	91.2	214.8	1478.0	2.6	17.6	15.1
Nod-004	203.4	43.9	12.3	15.1	67.7	105.8	751.1	1.2	10.3	13.3
Nod-011	445.6	89.7	28.7	19.9	172.5	215.2	2232.7	3.2	21.2	18.6
Nod-013	935.5	238.8	43.3	33.7	207.8	400.3	2567.7	4.4	38.2	27.6
Nod-014	1100.7	213.1	96.3	43.2	146.4	490.3	2729.0	4.8	37.9	24.8
Nod-016	449.7	107.7	32.0	21.7	132.1	184.9	1920.0	2.8	21.1	18.5
Nod-017	703.7	146.9	42.6	22.2	112.4	226.6	1407.6	2.7	26.3	19.1
Nod-021	1178.7	260.8	56.7	54.9	235.7	652.0	4684.2	7.1	39.2	28.2
Nod-022	1041.6	228.9	48.6	36.1	295.7	448.7	4004.1	6.1	39.6	26.5
Nod-024	1299.5	250.5	65.3	59.0	231.4	747.1	4252.3	6.9	43.5	28.9
*Mean*	799.9	172.0	48.1	31.8	169.3	368.6	2602.6	4.2	29.5	22.0
Cri-01	–	138.7	–	3828.1	54.1	179.8	23.0	4.2	1.9	12.6
Cri-02	–	149.0	–	4815.7	27.3	319.6	–	5.3	2.5	17.4
*Mean*	–	143.8	–	4321.9	40.7	249.7	11.5	4.8	2.2	15.0
Grav-01	511.0	287.8	33.9	2742.5	–	3593.3	32.6	7.2	7.5	12.7
Grav-02	148.9	128.0	36.2	2695.3	–	5058.0	–	8.1	8.7	13.9
*Mean*	329.9	207.9	35.0	2718.9	–	4325.6	16.3	7.6	8.1	13.3
MS										
*species*	1.3⋅10^6^	2.1⋅10^3^	2.9⋅10^2^	1.9⋅10^7^	2.8⋅10^4^	1.3⋅10^7^	9.6⋅10^6^	9.9	8.6⋅10^2^	90.6
*residual*	1.3⋅10^3^	5.9⋅10^3^	4.7⋅10^2^	4.5⋅10^4^	4.8⋅10^3^	1.4⋅10^5^	1.4⋅10^6^	3.4	1.1⋅10^2^	28.5
P. *F-* test	0.022	0.712	0.454	<0.001	0.037	<0.001	0.013	0.098	0.007	0.082

**Notes.**

a Samples Nod-003 to Nod-024 refer to the ten samples of species fool’s watercress evaluated. Samples Cri-01 and Cri-02 refer to the two samples of parsley evaluated. Samples Grav-01 and Grav-02 refer to the two samples of celery evaluated.

TITLE CF der caffeic acid derivatives CMA *p-*coumaric acid FRA ferulic acid AP apigenin KM kaempferol LT luteolin QR quercetin

**Figure 3:**
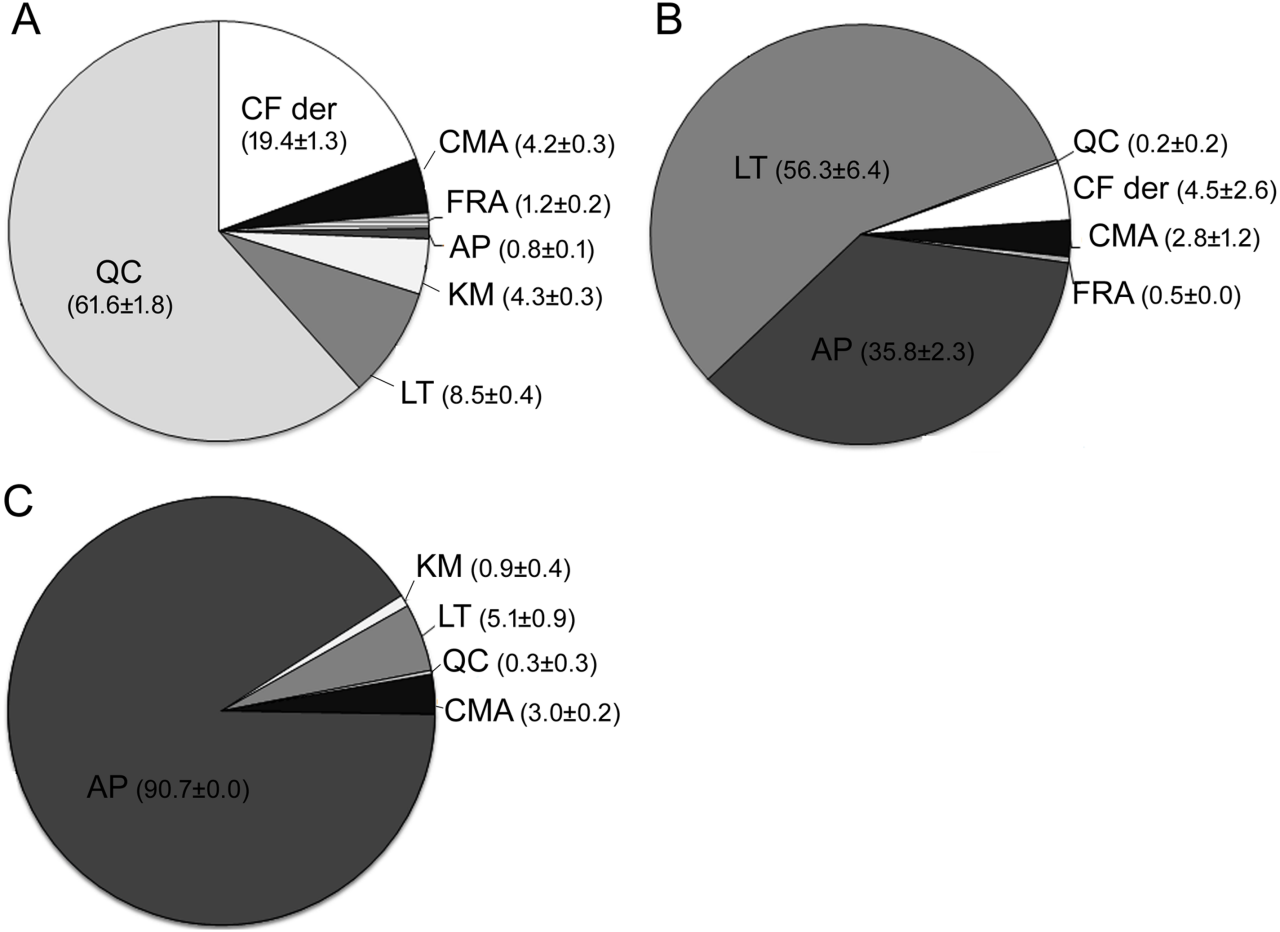
Relative abundance and standard error (µg compound µg^−1^ total) of individual phenolics identified. (A) Phenolics identified in the samples of fool’s watercress. (B) Phenolics identified in the samples of celery. (C) Phenolics identified in the samples of parsley. The abbreviations correspond to: caffeic acid derivatives (CF der), *p-* coumaric acid (CMA), ferulic acid (FRA), apigenin (AP), kaempferol (KM), luteolin (LT) and quercetin (QR).

In addition, the relative content of each compound against the sum of the phenolics targeted was determined (%, indicating mg of each compound mg^−1^ of total compounds) (Fig. 3). In general, flavonoid compounds comprised the most representative group considering the total identified, with an average relative abundance of 76.4%, 92.7% and 97.0% for fool’s watercress, celery and parsley, respectively. Nevertheless, the profile of individual phenolics varied considerably between species, both qualitatively and quantitatively. Quercetin was the major flavonoid in fool’s watercress (61.6%), while this flavonoid represented less than 0.3% in celery and parsley. On the contrary, apigenin was found as the major phenolic in parsley (90.7%) but it only represented 0.8% in fool’s watercress. This flavonoid ranked second in concentration in celery (35.8%), after luteolin (56.3%). Compared to celery, luteolin abundance was 6.6-fold lower in fool’s watercress and 11-fold lower in parsley. Finally, kaempferol was present in fool’s watercress in a relative abundance of 4.3% in contrast with parsley, in which represented only 0.9%. This compound was not detected in celery.

Differences in the composition of phenolic acids were also determined. The only hydroxycinnamic acid detected in leaves of parsley was *p-*coumaric and represented 3.0% of the total phenolics. On the contrary, the four hydroxycinnamic acids targeted were detected in fool’s watercress and celery. The relative concentration of these compounds in leaves of celery ranged from 0.5% for ferulic acid to 4.5% for caffeic acid derivatives. Ferulic acid was also the minor phenolic acid detected in fool’s watercress (1.2%), followed by *p-*coumaric acid (4.2%). Caffeic acid derivatives were determined as the main hydroxycinnamic acids of fool’s watercress (19.4%).

### Correlation between antioxidant parameters

The Pearson linear correlation coefficient (r) between the DPPH radical-scavenging activity and TPC in fool’s watercress populations was *r* = 0.903 (*P* < 0.001). For those samples of fool’s watercress that were analysed by HPLC, correlation coefficient values between DPPH, TPC and the content in targeted phenolics were also studied. The content in targeted phenolics as sum of the individual compounds presented high correlation coefficients with both the DPPH scavenging activity and TPC (*r* = 0.924 and *r* = 0.933, respectively; *P* < 0.001).

## Discussion

The present study highlights the antioxidant capacity of fool’s watercress in terms of DPPH scavenging activity and TPC. These results are in agreement with published data. For example, Morales et al. (2012) analysed four wild leafy vegetables (*A. nodiflorum, F. vulgare*, *Montia fontana* L. and *Silene vulgaris* (Moench) Garcke.) and obtained the highest values for DPPH and TPC in fool’s watercress. In addition, fool’s watercress also has high antioxidant activity compared to other common aromatic herbs and spices from the same family. Hossain et al. (2011) evaluated those parameters for fennel, celery, cumin and parsley. Values of DPPH-radical scavenging activity in these spices were 1.7 to 9.7-fold lower than the antioxidant capacity of fool’s watercress. On the contrary, the TPC calculated there for the four spices were quite similar to the range determined in fool’s watercress.

Comparison among different geographical groups of fool’s watercress revealed moderate differences for the TPC and the DPPH radical scavenging activity. The production and accumulation of these secondary metabolites can be affected by the environmental conditions and stress situations as well as by genetic diversity (Kaulmann et al., 2014; Galieni et al., 2015). Thus, differences between geographical groups may result from genotypic differences or by divergence of particular environmental conditions. The evaluation of genetic and environmental effects needs to be studied in future selection programmes aimed at the development of fool’s watercress as a new crop with high antioxidant properties as added value.

Our results also reveal differences in the phenolic profile of the three species. Fool’s watercress displayed low amounts of apigenin and luteolin in contrast to parsley and celery, vegetables described as sources of these compounds (Zhou et al., 2016). On the contrary, quercetin was detected as the main flavonoid of fool’s watercress. Quercetin has been also detected within the *Apiaceae* in fresh herbs such as coriander, dill or fennel (Barros et al., 2012; Salami, Rahimmalek & Ehtemam, 2016; El-Zaeddi et al., 2017). Regarding to the hydroxycinnamic acids studied (caffeic acid, chlorogenic acid, *p*-coumaric acid and ferulic acid), the four of them have been previously detected in fennel (Salami, Rahimmalek & Ehtemam, 2016); these authors found that chlorogenic acid was the main phenolic acid. Chlorogenic acid is rapidly hydrolysed to caffeic acid in alkaline conditions (Mattila & Kumpulainen, 2002), while it is considered more stable at low pH. However, we noted a partial hydrolysis of chlorogenic acid to caffeic acid under the current conditions. Due to this reason, we considered both hydroxycinnamic acids, that is, chlorogenic and caffeic acids, together as caffeic acid derivatives, and the individual values were not given.

In agreement with our results, positive, strong correlation between DPPH and TPC has been previously established in other vegetables, including common spices and wild species (Morales et al., 2014; Skotti et al., 2014; Tang et al., 2015). However, in other cases this correlation was not so clear (Albano & Miguel, 2011). Discrepancies may be explained in part by the composition of the evaluated matrix, as well as the possible interferences of different compounds others than phenolics with the reagents, as they could be tocopherols, aminoacids or, commonly to many fruits, ascorbic acid (Craft et al., 2012). However, the ascorbic acid is an unstable metabolite highly sensitive that can be easily degraded with the consequence of losing the antioxidant capacity. Conditions such as exposure to oxygen (e.g., during the extraction step and storage of extracts), humidity, or temperature of drying and store can affect the stability of ascorbic acid (e.g., Kaya, Aydin & Kolayli, 2010; Van Bree et al., 2012). Moreover, this molecule is commonly stabilized with *meta*-phosphoric, in order to preserve it from degradation during storage of the extract (Chebrolu et al., 2012). On the contrary, phenolic compounds are stable molecules, not affected by the drying process (Bianchi & Lo Scalzo, 2018). Our results suggest that the antioxidant capacity of the dried leaves in this species is mainly due to the phenolic compounds, according to the high correlation established between the two parameters. In the same way, the high correlation between the sum of the individual phenolics of fool’s watercress detected by HPLC and both the DPPH radical-scavenging activity and TPC indicated that the compounds identified account for the antioxidant activity of the species.

Although celery presented the highest values of phenolics measured by HPLC and contents determined in parsley were also remarkable, the antioxidant capacity measured in these species was lower than the obtained in fool’s watercress, especially in the case of DPPH radical-scavenging activity. A possible reason may be found in the chemical structure of the major compounds detected in the different species, as well as the synergistic effects of different phenolic compounds present in specific species. The number and position of hydroxyl groups affect the antioxidant capacity of polyphenols (Cartea et al., 2011; Zaluski, Ciesla & Janeczko, 2015). The antioxidant capacity of these compounds would decrease in the following order: quercetin >luteolin >apigenin (Yildiz et al., 2008), which could explain the relatively poor DPPH activity of parsley in comparison with the two other species. In celery, both apigenin and luteolin would account for an important share of the antioxidant capacity. Finally, the highly remarkable DPPH radical-scavenging activity of fool’s watercress would be correlated to the content in quercetin, which is in addition a molecule that has been related with a protective and inhibition action against several cancers, in cellular models but also *in vivo* in mammals (Sharmila et al., 2014).

## Conclusions

Our results reveal that fool’s watercress is a leafy vegetable with high antioxidant activity, especially in comparison to the related cultivated parsley and celery. The high correlation between DPPH radical scavenging activity and TPC suggested that the antioxidant activity of this species is mainly caused by the phenolic compounds accumulated in the leaves. When the phenolic profile was analysed, we observed that, unlike celery and parsley, quercetin was the main compound present in the species. This finding may explain the greatest antioxidant activity of fool’s watercress, resulting from the higher antioxidant capacity of this flavonoid compared to apigenin and luteolin, the main compounds detected in parsley and celery, respectively (Rice-Evans, Miller & Paganga, 1996). In addition, results revealed differences among the geographical groups established for the total of populations of fool’s watercress, indicating that selection among geographical origins may result in differences in bioactive properties. Although these differences may be caused by either genetic variation or environmental conditions, our results offer a starting point for future domestication and breeding programs.

##  Supplemental Information

10.7717/peerj.6296/supp-1 Figure S1 Representative chromatograms of fool’s watercress, celery and parsley (A) Chromatogram obtained with the conditions described by Yildiz et al. (2008), and identification of the hydroxycinnamic acids targeted. Peaks correspond to: 1 chlorogenic acid, 2 caffeic acid, 3*p*-coumaric acid, and 4 ferulic acid. (B) Chromatogram obtained with the conditions described by Bae et al. (2012), and identification of the flavonoids (aglycones) targeted. Peaks correspond to: 5 quercetin, 6: luteolin, 7: kaempferol, and 8: apigenin. Note that different chromatograms may have different scales.

10.7717/peerj.6296/supp-2 Data S1 Raw data of the analyses performed Raw data file includes the content in total phenolics (mg of chlorogenic acid equivalents per gram of dry matter), and the DPPH value (mg of Trolox equivalents per gram of dry matter), for the twenty-five populations of fool’s watercress (Nod-001 to Nod-025), two of parsley (Cri-01 and Cri-02), and two of celery (Grav-01 and Grav-02). The file also includes the content of individual phenolics (micrograms per gram of dry matter) targeted by HPLC for the selected samples. Each data correspond to one individual measurement.

## Additional Information and Declarations

### Data Availability

The following information was supplied regarding data availability: The raw data is available in the Supplemental File.
