# Supplementary figures and images for: Wild edible fool’s watercress, a potential crop with high nutraceutical properties
Source: PeerJ. 2019 Feb 1;7:e6296. doi: 10.7717/peerj.6296 (PMC6361001; doi:10.7717/peerj.6296)

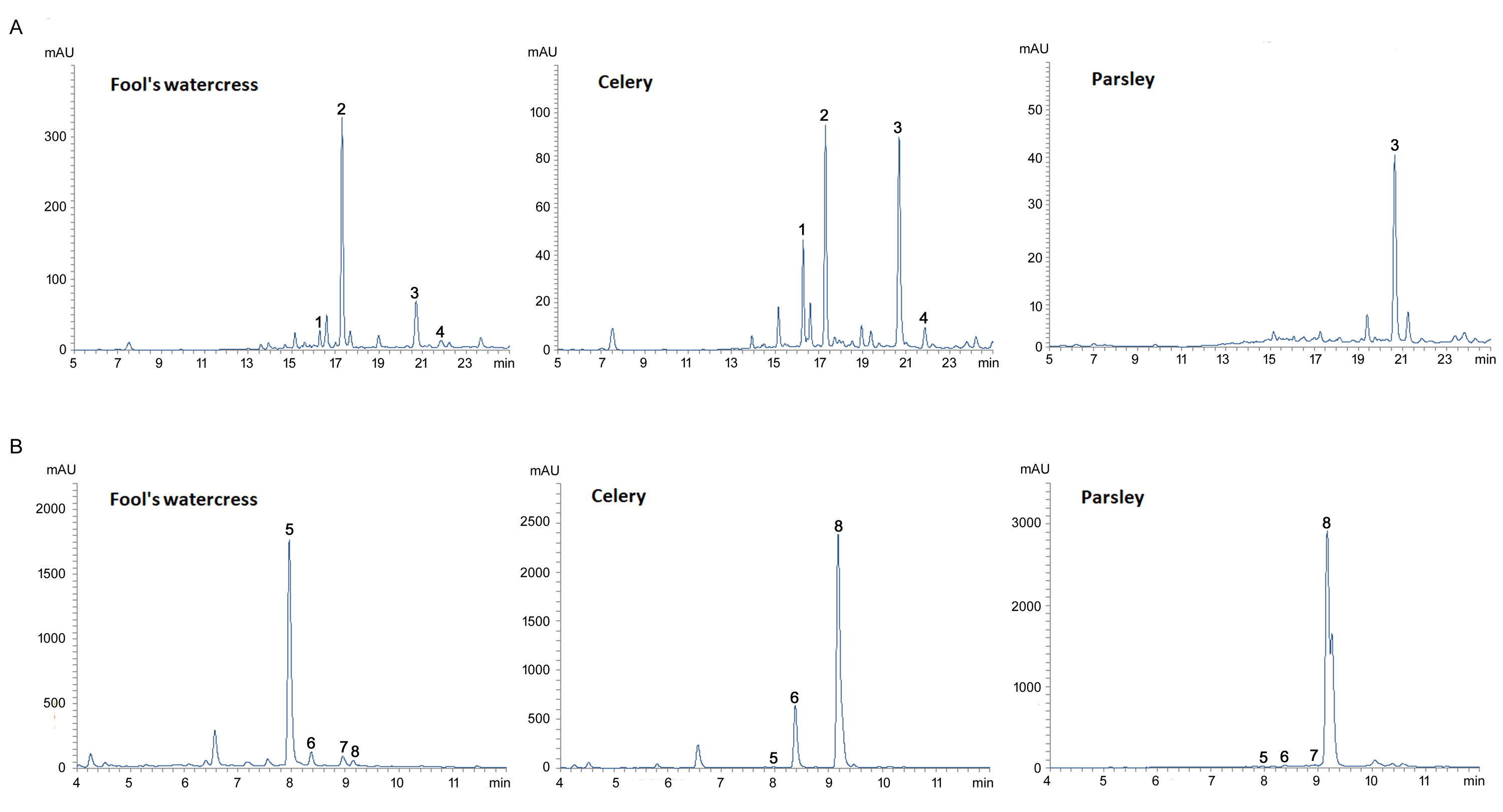

Supplement: Figure S1 — (A) Chromatogram obtained with the conditions described by Yildiz et al. (2008), and identification of the hydroxycinnamic acids targeted. Peaks correspond to: 1 chlorogenic acid, 2 caffeic acid, 3p-coumaric acid, and 4 ferulic acid. (B) Chromatogram obtained with the conditions described by Bae et al. (2012), and identification of the flavonoids (aglycones) targeted. Peaks correspond to: 5 quercetin, 6: luteolin, 7: kaempferol, and 8: apigenin. Note that different chromatograms may have different scales. [file peerj-07-6296-s001.png]
